# Secreted Phosphoprotein 24 kD (Spp24) and Spp14 Affect TGF-β Induced Bone Formation Differently

**DOI:** 10.1371/journal.pone.0072645

**Published:** 2013-08-26

**Authors:** Haijun Tian, Xiaoda Bi, Chen-Shuang Li, Ke-Wei Zhao, Elsa J. Brochmann, Scott R. Montgomery, Bayan Aghdasi, Deyu Chen, Michael D. Daubs, Jeffrey C. Wang, Samuel S. Murray

**Affiliations:** 1 Department of Orthopaedic Surgery, Changzheng Hospital, Second Military Medical University, Shanghai, P. R. China; 2 Department of Ophthalmology, the 309th Hospital of PLA, Beijing, P. R. China; 3 Department of Orthodontics, Peking University School and Hospital of Stomatology, Beijing, P. R. China; 4 Research Service, Education and Clinical Center, VA Greater Los Angeles Healthcare System, North Hills, California, United States of America; 5 Geriatric Research, Education and Clinical Center, VA Greater Los Angeles Healthcare System, North Hills, California, United States of America; 6 Department of Medicine, University of California Los Angeles, Los Angeles, California, United States of America; 7 Department of Physiology, University of California Los Angeles, Los Angeles, California, United States of America; 8 Department of Orthopaedic Surgery, University of California Los Angeles, Los Angeles, California, United States of America; 9 Department of Orthopaedic Surgery, the Methodist Hospital System, Houston, Texas, United States of America; Northwestern University, United States of America

## Abstract

Transforming growth factor-β (TGF-β) and bone morphogenetic proteins (BMPs) have opposing but complementary functions in directing bone growth, repair, and turnover. Both are found in the bone matrix. Proteins that bind to and affect the activity of these growth factors will determine the relative abundance of the growth factors and, therefore, regulate bone formation. Secreted phosphoprotein 24 kD (Spp24) is a bone matrix protein that has been demonstrated to bind to and affect the activity of BMPs. The arginine-rich carboxy terminus of Spp24 is proteolytically processed to produce three other predictable truncation products (Spp18.1, Spp16.0, and Spp14.5). In this work, we report that kinetic data obtained by surface plasmon resonance demonstrate that Spp24 and the three C-terminal truncation products all bind to TGF-β1 and TGF-β2 with a similar but somewhat less affinity than they bind BMP-2; that, as in the case of BMP-2, the full-length (FL) form of Spp24 binds TGF-β with greater affinity than do the truncation products; that FL-Spp24 inhibits TGF-β2 induced bone formation *in vivo*, but Spp14.5 does not; and that co-administration of FL-Spp24 or Spp14.5 with TGF-β2 *in vivo* is associated with a reduction in the amount of cartilage, relative to new bone, present at the site of injection. This finding is consistent with the observation that low-dose TGF-β administration *in vivo* is associated with greater bone formation than high-dose TGF-β administration, and suggests that one function of Spp24 and its truncation products is to down-regulate local TGF-β activity or availability during bone growth and development. The similarities and differences of the interactions between Spp24 proteins and TGF-β compared to the interaction of the Spp24 proteins and BMPs have significant implications with respect to the regulation of bone metabolism and with respect to engineering therapeutic proteins for skeletal disorders.

## Introduction

Mature, mineralized bone contains a number of growth factors that are essential for proper bone remodeling and repair [1]. Among these growth factors, bone morphogenetic proteins (BMPs), especially BMP-2 and -7, and transforming growth factor-β (TGF-β), are the most significant. These regulatory molecules have complementary but also opposing activities. In general, TGF-β enhances preosteoblast proliferation and extracellular matrix synthesis but also opposes the BMP effect on osteoblast differentiation [2,3]. On the other hand, recombinant BMPs such as BMP-2 and -7 enhance osteoblast differentiation [4] but are also capable of inducing the entire recapitulation of endochondral bone formation as originally described by Urist [5]. This process involves stem cell proliferation, chondrogenic differentiation, and replacement of cartilage by bone. TGF-β and BMPs (BMP-2 and -7) are present in bone matrix in similar concentrations [6,7]. Bone also contains a number of extracellular proteins that bind to members of the TGF-β superfamily of cytokines and regulate their activity [8–10]. TGF-β has a family of associated proteins that maintain the active molecule in the matrix in a latent form [11]. No such group of proteins has yet been described in relation to the BMPs.

One matrix protein that has binding affinity for several members of the TGF-β family of proteins is secreted phosphoprotein 24 kD (Spp24) [10]. This liver-derived bone matrix protein is exquisitely labile to proteolysis [12] and circulates in a protective complex with α-macroglobulins and anti-thrombin III (Serpin C1) [13]. The protein exists in the bone environment in several forms ranging in size from 14 kD to 24 kD [14] with a dominant isoform of about 18.5 kD [15]. Degradation of recombinant Spp24 gives rise to well defined proteins of 18.1 kD, 16.0 kD, and 14.5 kD [12]. These degradation products retain the N-terminus of the mature parental protein and have a truncated C-terminus of various lengths. When co-implanted with BMP-2 in different models of BMP-induced bone formation, Spp24 and its derivatives inhibit bone formation. Significantly, the full-length molecule is more inhibitory than the truncated forms [16].

We hypothesized that TGF-β would bind to Spp24 and its derivatives in a manner similar to that in which Spp24 binds to BMP-2 and that Spp24 and its proteolytic derivatives would inhibit TGF-β activity to different degrees. In the present study, we have tested that hypothesis and confirmed that Spp24 and its C-terminal truncation products bind TGF-βs and modulate their bioactivity. Therefore, it is likely that the bone matrix protein Spp24, which influences the activity of both BMPs and TGF-β, plays a significant role in the overall control of the BMP/TGF-β economy of the bone environment and that proteolysis of Spp24 is one mechanism through which more refined levels of control are imparted to this mechanism.

## Materials and Methods

### Materials

Recombinant human BMP-2, human TGF-β1, and human TGF-β2 were purchased from R & D Systems (Minneapolis, MN). Recombinant Spp24 proteins were produced in a bacterial expression system and purified by IMAC (immobilized metal affinity chromatography) chromatography using a BioLogic chromatography workstation (BioRad, Hercules, CA) as described in detail previously [12].

### Surface Plasmon Resonance

Surface plasmon resonance analyses of protein interactions were performed on a Biacore T-100 instrument (G.E. Healthcare, Piscataway, NJ). CM5 chips, HBS-EP running buffer and amine coupling reagents were obtained from the manufacturer. BMP and TGF-β were immobilized as the ligands whereas the four Spp24 proteins were employed as the analytes. In order to obtain precise measurements of the concentration of the analytes each Spp24 protein was dissolved in water at a concentration of 1 mg/ml, mixed thoroughly, centrifuged at 12,000 x g for one minute, and then decanted. Concentrations were determined using custom coefficients obtained from the ProtParam tool of ExPASy (web.expasy.org; Swiss Institute of Bioinformatics, Lausanne, CH). In calculating the coefficients, it was assumed that one half of the cysteine residues were oxidized. The coefficients (in the form of X units of absorption at 280 nmeter = 1g/L) where: X= 1.267 for Spp24, X= 1.127 for Spp18.1, X= 1.161 for Spp16.0, and X= 1.189 for Spp14.5. Five concentration of each analyte were tested with each ligand. Kinetic constants were calculated using software supplied by the manufacturer.

### In vivo bioassay

All research involving animals was reviewed and approved by the VA Greater Los Angeles Healthcare System Institution Animal Care and Use Committee (IACUC) prior to the initiation of any experiments. Research on living, non-human subjects at this institution is in compliance with the guiding principles in the Guide for the Care and Use of Laboratory Animals (Eighth Edition). Four-day-old Long Evans rats were divided into four groups and received 14 daily injections of test materials. Group I received 10 μl injections of TGF-β2 50 ng; Group II received 10 μl injections of TGF-β2 50 ng and Spp24; Group III received 10 μl injections of TGF-β2 50 ng and Spp14.5; Group IV received 10 μl PBS only. Proteins were dissolved in PBS, pH 7.4. The quantities of Spp24 and Spp14.5 were devised to be equimolar at 11.5 μM (the highest common solubility that could be achieved for the two proteins) and to provide a molar ratio of about 29 fold molar excess with respect to TGF-β2.

Injections were performed following a modification of the protocol of Joyce, et al. [17]. A dull 27-gauge needle was used for the subperiosteal injection technique. Microinjections were directed into the subperiosteal region of the anterior-superior surface of the rat femur. This technique was perfected by injecting dye until injections could be consistently reproduced. All the rats were injected on the left femurs only for 14 consecutive days, and right femurs were left intact to serve as their own control to quantify new bone formation. Femurs were harvested on the 15^th^ day after receiving 14 injections. An incision was made on the superior skin along the femur and the hip joint, and the knee joint were exposed. All ligaments were dissected away so that the femoral head could be taken out of the acetabulum and then the femur was also separated from the tibia and fibula. Great caution was taken when removing the femur to preserve the intact bone. Some muscle tissue attached to the femur was left in place because of the possibility of bone formation extending into the muscle.

### Radiographic analyses

X-rays were taken using a Faxitron small specimen X-ray cabinet (Faxitron; Tucson, AZ) with an operating voltage of 35 KV and an exposure time 12.5 s. The area of the mass of new bone formation apparent on the radiographs was measured for each subject using ImageJ software (National Institutes of Health; http://rsb.info.nih.gov/ij/).

### Micro-computerized tomography (μCT)

Rat femurs were analyzed by high resolution micro-computed tomography (µCT), using a µCT imaging system (µCT40, Scanco Medical AG, Brüttisellen, Switzerland) with a resolution of 16µm and an X-ray energy of 55kVp and 160mA, calibrated against a hydroxyapatite (HA) phantom. Five hundred projections were acquired per 180-degree rotation with an integration time of 300 ms. A threshold of 122 was used to discriminate bone and soft tissue. The entire femur was included for every scan. Left femurs were analyzed for bone formation and the right femur from the same animal subject was scanned and used as a control. New bone formation volume (BV) was calculated by subtracting the volume of the right femur from the volume of the left femur. A 2D contouring algorithm was used to identify the new bone formation area in the rat femurs in TGF-β group and TGF-β + Spp14 group, and relative bone volume (BV/TV) was calculated using the same software. The BV/TV value was not calculated in the PBS and TGF-β + Spp24 group because there were not enough new bone and it was difficult to identify the new bone formation area.

### Histology

After imaging was completed, the specimens were fixed in 10% formalin, decalcified, washed with tap water, and then transferred to 70% ethanol. Serial longitudinal sections were carefully cut at the site of new bone formation. Specimens were embedded in paraffin, sectioned, deparafﬁnized in xylene, hydrated in graduated ethanols, and stained with hematoxylin and eosin (H&E), Safranin O and Alcian Blue. Safranin O sections were stained in 0.2% Fast Green (Sigma, St Louis, MO, USA), for 5 min, pretreated with 1% acetic acid and then stained in 0.1% Safranin O stain for 1 hour and then counterstained with hematoxylin. Alcian Blue sections were stained with 1% Alcian Blue at pH 2.5 for 30 minutes, thoroughly rinsed with tap water, and then counterstained with nuclear fast red for 5 min.

### Numerical analysis

The area of the mass of new bone formation apparent on the radiographs was measured for each subject. Comparisons of means of groups by either ANOVA or Student’s *t*-test were conducted using IBM SPSS Statistics (version 20, IBM, Armonk, NY).

## Results

**Figure 1:**
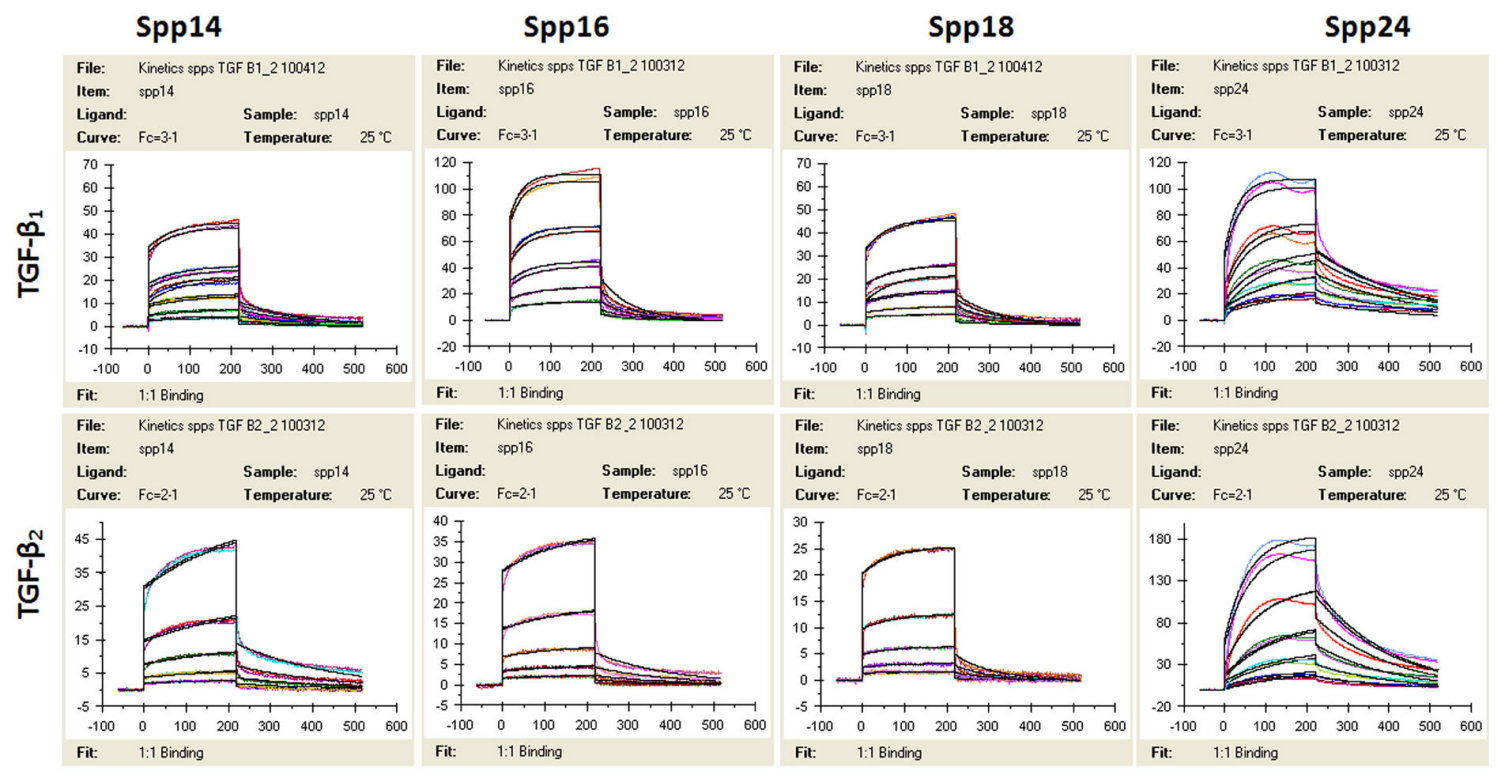
Sensogram from the surface plasmon resonance analysis of the interaction between TGF-β1, β2 and different Spps. The response (RU or response units) is plotted on the Y-axis, while time (seconds) is plotted on the X-axis.

The kinetics of the binding of FL-Spp24 and its degradation products to TGF-β1 and -β2 were determined by surface plasmon resonance (Figure 1, Table 1), and the results are compared to those obtained with recombinant human BMP-2, which were previously published [15,18]. Both TGF-β1 and -β2 bound each of the four Spp24 proteins (FL-Spp24, Spp18.1, Spp16, and Spp14.5). Kinetic analysis demonstrated that FL-Spp24 had the greatest affinity in both cases, and the affinity of binding by C-terminally truncated Spp24 derivatives was one to two orders of magnitude lower than that of FL-Spp24. Qualitatively similar results were obtained for the binding to BMP-2, although the affinity for BMP was about an order of magnitude higher than the affinity for TGF-βs [18]. Thus, Spp24 and its C-terminal truncation products bind human TGF-β1 and –β2 with lower affinity than they bind recombinant human BMP-2, and C-terminal truncation of Spp24 reduces its binding affinity. Given the relatively equivalent concentrations of TGF-βs and BMPs in bone [6,7], the kinetics of binding suggest that Spp24 and its degradation products would bind BMPs at lower concentrations than TGF-βs, thus achieving a relative sequestration of BMPs when compared to free TGF-βs.

**Table 1:** Kinetic parameters as determined by surface plasmon resonance for the interactions of TGF-β1, TGF-β2, and BMP-2 versus each of the four Spp24 isoforms.

		spp14.5	spp16.0	spp18.1	spp24
TGF-β1	k_a_ (M^-1^ s^-1^×10^3^)	4.18	5.55	10.00	71.9
	k_d_ (s^-1^×10^-3^)	5.89	12.2	10.6	3.93
	K_D_ (k_d_//k_a_) (μM)	***1.41***	***3.25***	***1.39***	***0.065***
TGF-β2	k_a_ (M^-1^ s^-1^×10^3^)	2.39	1.34	3.64	32.4
	k_d_ (s^-1^×10^-3^)	5.39	5.66	10.9	5.86
	K_D_ (k_d_//k_a_) (μM)	***3.22***	***4.24***	***3.18***	***0.194***
BMP-2	ka (M^-1^ s^-1^×10^3^)	15.3	63.6	263	311
	k_d_ (s^-1^×10^-3^)	3.60	2.61	1.26	5.50
	K_D_ (k_d_//k_a_) (μM)	***0.236***	***0.041***	***0.005***	***0.018***

k_a_ represents “recognition”, k_d_ represent “stability”, and K_D_ stand for the actual “affinity” of the proteins. The values pertaining to BMP-2 are from reference 16 and are shown for purposes of comparison.

**Figure 2:**
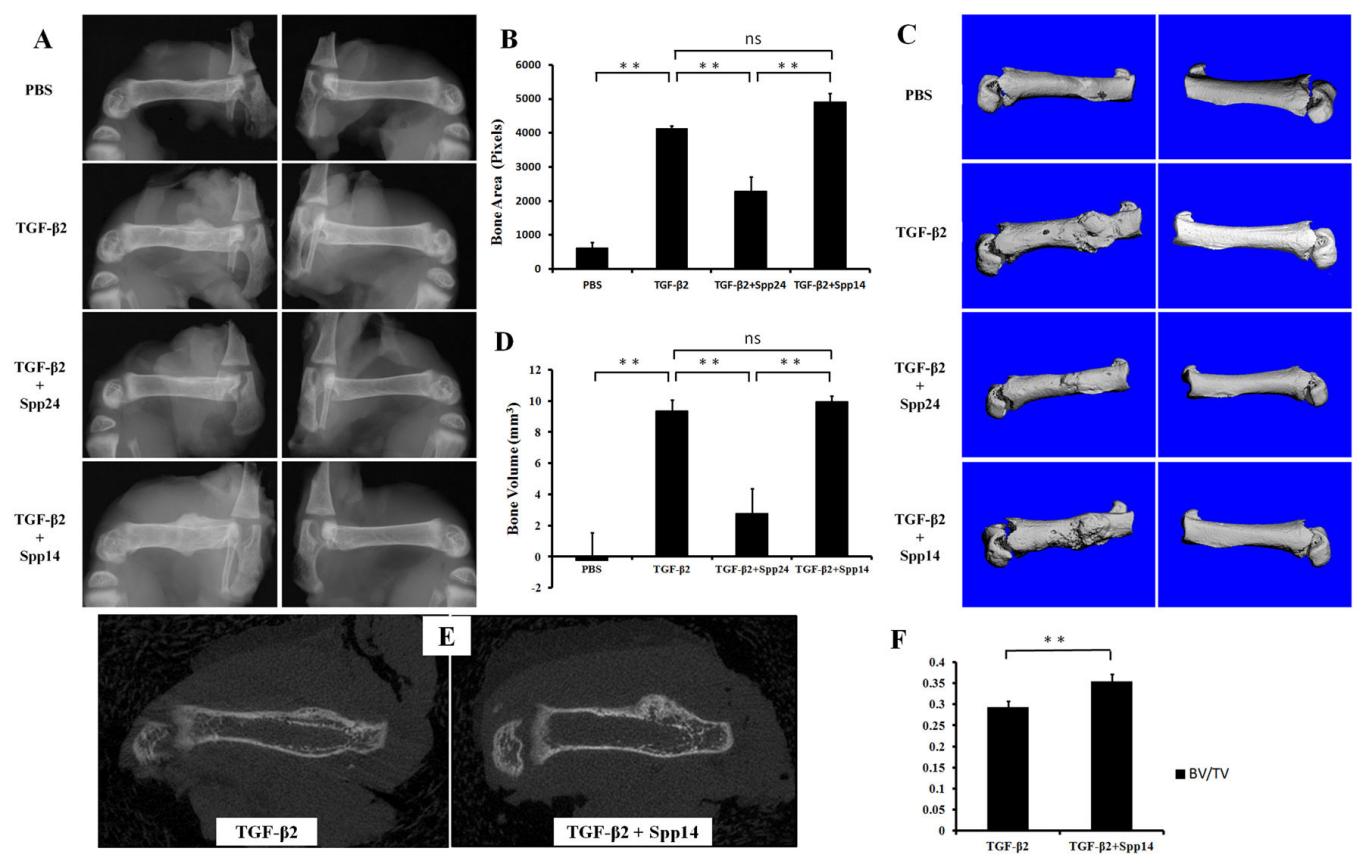
Radiographs of femurs of newborn rats treated for 14 consecutive days with TGF-β2 alone or with Spp24 or with Spp14.5. Representatives of treatment groups as follows: A.X-ray images, with both various treated left femurs and untreated right femurs; B. Bone area derived from radiographic analysis of femurs of newborn rats with different treatment (**: P<0.01); C. CT images of treated femurs and untreated control; D. Bone volume of newly formed bone derived from CT images (**: P<0.01); E. 2D CT image comparing new bone quality of TGF-β2 group and TGF-β2 + Spp14.5 group; F. BV/TV value from the new bone formation area of TGF-β2 group and TGF-β2 + Spp14.5 group.

**Figure 3:**
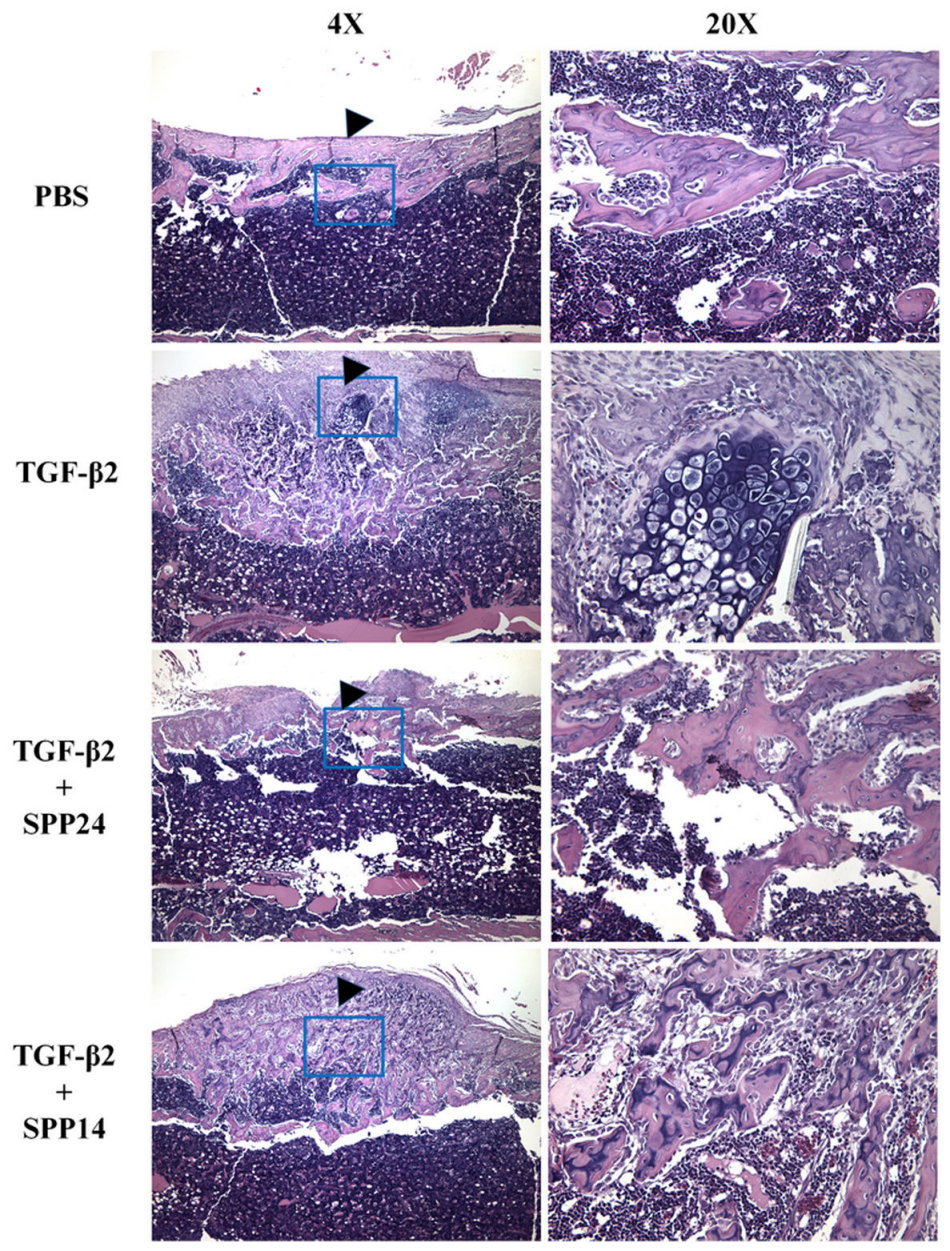
Histologic examination of representative samples from each experimental groups. Stained with H&E.

**Figure 4:**
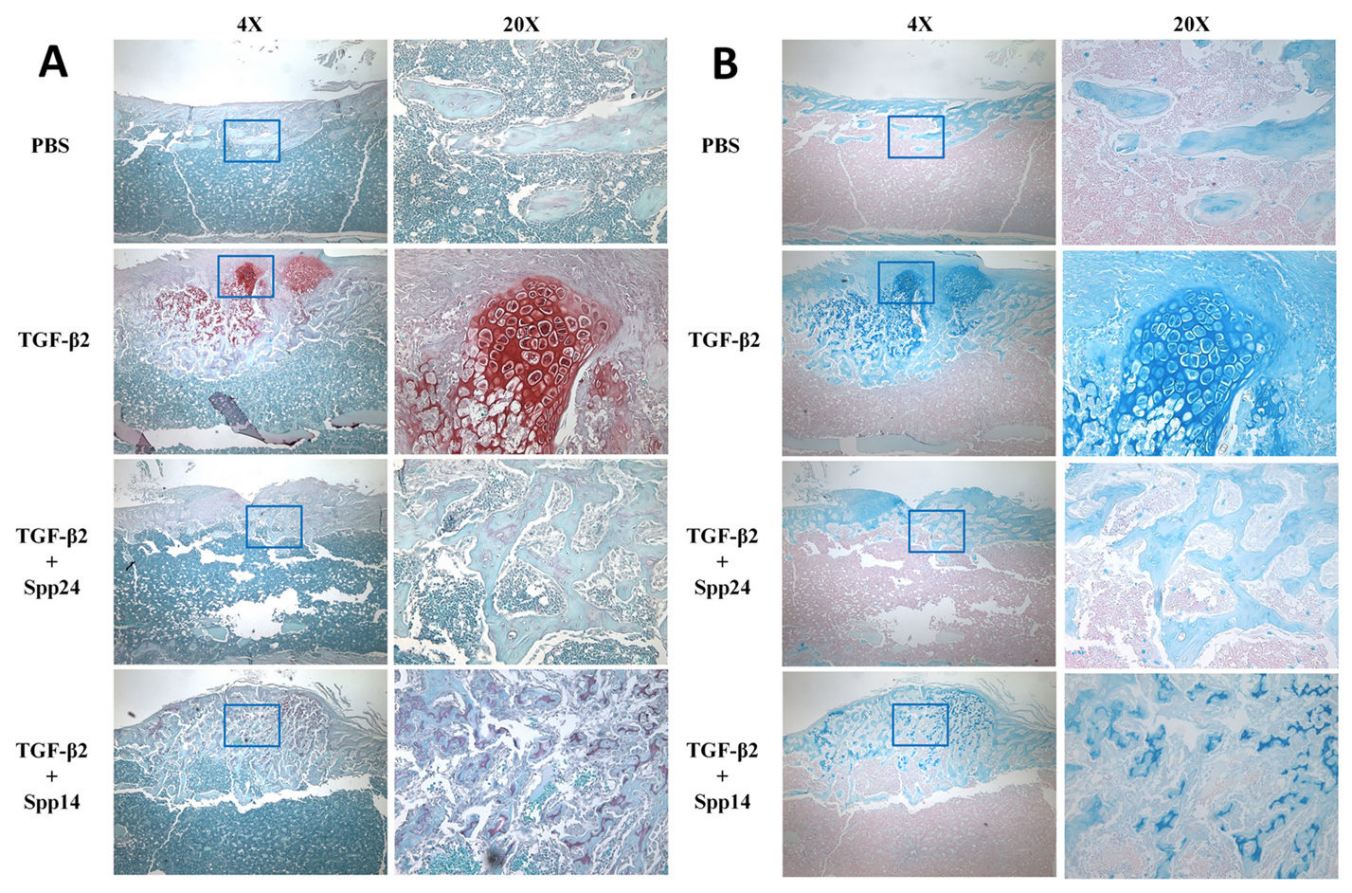
Histological examination of specimens from each treatment groups stained with Alcian Blue or Safranin O. Note that Spp24 and Spp14.5 decrease the amount of cartilage-specific staining in TGF-β2-treated bone.

Having established that Spp24 and its derivatives bind TGF-β1 and -β2 with high affinity, we then assessed the biological effects of Spp24 and Spp14.5 on TGF-β2-induced bone formation in newborn rats. All of the rats grew well after the injections and no nerve injury, vascular injury, or other complications were observed. Radiographs of representative subjects taken after completion of the injections are shown in Figure 2. Visual inspection of X-rays confirmed that all of the limbs that received an injection of TGF-β2 alone demonstrated obvious new bone formation whereas none of the limbs in the PBS only control group showed any new bone formation (Figure 2, Panel A). These results are consistent with those reported by Joyce, et al. [17]. The area of the mass of new bone formation apparent on the radiographs was measured (Figure 2, Panel B). The new bone formation in the PBS alone group was very low, while significant bone formation as seen in the TGF-β2 group. The bone forming activity of TGF-β2 was inhibited by Spp24 as manifest by a mass area about half of that of TGF-β2 alone. Interestingly, Spp14.5 did not show the same inhibitory effect as did Spp24, but rather was associated with a modest increase in bone formation above TGF-β2-treated levels that was not statistically significant. Analyses of the images from μCT confirmed the results obtained from the X-rays (Figure 2, Panels C and D). Average new bone volume and BV/TV for each of the groups was shown in Table 2. The relative bone volumes of the TGF-β2 group and TGF-β2 + Spp14.5 groups were also measured (Figure 2, Panels E and F), showing that TGF-β2 + Spp14.5 group had a higher BV/TV value. Figure 3 shows histological sections from specimens representing each group. This analysis confirmed that the masses on the femurs from animal subjects from the treatment groups were composed of bone and cartilage. Only minimal trauma was visible in the PBS treatment group. A paucity of cartilage was observed in specimens from the TGF-β2 plus Spp14.5 group, which was confirmed in histologic sections stained for cartilage with Safarin oil red O and for sulfated proteoglycans synthesized by functional chondrocytes with Alcian blue (Figure 4, Panels A and B). This explains why the TGF-β2 plus Spp14.5 group has a higher BV/TV volume than TGF-β2 group.

**Table 2:** New bone volume (BV) and relative bone volume (BV/TV) of different groups.

	BV (mm^3^)	BV/TV
PBS	-0.263 ± 1.801	/
TGF-β2	9.370 ± 0.679	0.293 ± 0.015
TGF-β2+Spp24	2.784 ± 1.584	/
TGF-β2+Spp14	9.944 ± 0.376	0.355 ± 0.017

## Discussion

We hypothesized that Spp24 is an important regulator of the net balance of available free forms of the BMPs and TGF-β in skeletal tissue throughout the lifespan and initiated studies to define the physical interactions of Spp24 with BMP/TGF-β cytokines. We observed that Spp24 and its C-terminal truncation products bind human TGF-β1 and –β2 with lower affinity than they bind recombinant human BMP-2, and C-terminal truncation of Spp24 reduces its binding affinity. TGF-β induced bone formation was inhibited by FL-Spp24, but enhanced by Spp14.5. We also previously reported that FL-Spp24 inhibits BMP-2 and -7-stimulated bone formation, and C-terminal truncation reduces its inhibitory effects [19,20].

Given that Spp24 and its C-terminal truncation products bind BMP/TGF-β cytokines with different affinities, does this account for the differences in their biological effects?

**Figure 5:**
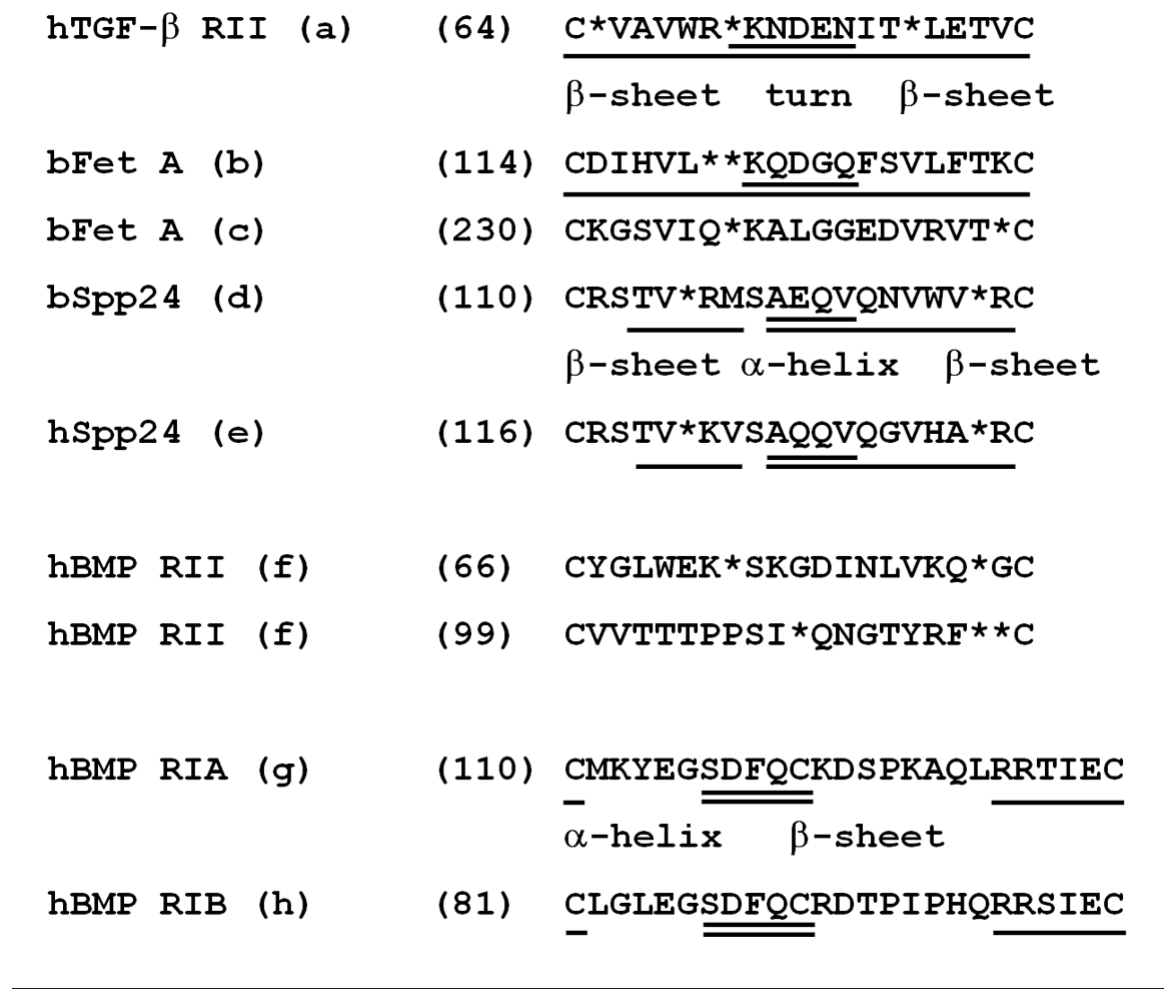
Sequence and secondary structure comparisons of various receptor and pseudoreceptor molecules proposed to give information pertaining to the site of interaction of Spp24 with BMP-2 and related cytokines. All sequences are from Unipro KB. The first residue number in the sequence of each protein is indicated in parentheses. Proposed secondary structural elements are indicated by underlining, with β-sheet domains underlined once, turn of α-helical domains underlined twice, and regions of undefined structure (“the rest”) receiving no underlining. Spaces between residues were inserted by the authors (or from references) to achieve alignments are indicated with an “*”. a. Human TGF-β receptor type II. P37173. Sequence selection, spacing, alignment, and structure assignment from Demetriou, et al. (21). Spacing modified by the authors. b. Bovine fetuin A. P12763. Sequence selection, spacing, alignment and structure assignment from Demetriou, et al. (21).. c. Bovine fetuin A “inactive” loop 2. Sequence selection, alignment and spacing by the authors. d. Bovine Spp24. Q27967. Sequence selection, alignment, spacing, and structure assignment by the authors. e. Human Spp24. Q13103. Sequence selection, alignment, spacing, and structure assignment by the authors. f. Human BMP receptor type II. Q13873. Sequence stated by Demetriou, et al. (21) but not specifically identified. Sequence selection, spacing and alignment by the authors. g. Human BMP receptor type IA. P36894. Sequence selection by the authors. Structure assignment by Kotzsch, et al. (27). h. Human BMP receptor type IB. O00238. Sequence selection by the authors. Structure assignment by Kotzsch, et al. (27).

The proposed mechanism for the interaction between BMP-2 and Spp24 is the presence of a domain in the middle of the Spp24 molecule (specifically amino acids 110 through 128 in the 203 amino acid bovine protein) [10] that shares some sequence similarity with the TGF-β type II receptor which would, presumably, bind to the same aspect of the BMP-2 molecule that is involved in physiological receptor binding. This, however, is far from certain. Demetriou, et al. [21] first pointed out the similarity of a region (the TRH1 or TGF-β receptor II homology-1 domain) in fetuin, which, like Spp24, contains a cystatin domain within which the areas with similarity to the TGF-β type II receptor are found. Cystatin domains are named for their similarity to the cysteine protease inhibitor, cystatin, but the domains in fetuin and Spp24 do not have any inhibition of proteolytic activity. Behnam, et al. [15] located in bovine Spp24 a region that is somewhat similar to the TRH1 domain in fetuin (Figure 5). The domains in both fetuin and Spp24 are 19 amino acids long and are bounded by a loop-forming disulfide bond between cysteine residues. The degree of similarity between the TRH1 domains in fetuin or Spp24 and the human TGF-β receptor type II (and related receptors) is, in actuality, quite small (Figure 5). The hypothesis of Demetriou, et al. [21] that the TRH1 domain was the binding site of TGF-β and BMP-2 to fetuin was based on sequence similarities to the TGF-β receptor type II and made no reference to functional studies such as epitope mapping which were, in fact, not available at the time. Subsequently, Guimond, et al. [22,23] produced an extensive series of point and deletion mutations in the corresponding region of the TGF-β type II receptor and reported that they had no effect on binding of TGF-β to the receptor. Similarly, Sun, et al. [24] reported that a number of the amino acids of the TRH1 domain of Spp24 (including the two terminal cysteines one at a time) could be substituted with alanine with minimal impact on the binding of the protein to BMP-2. Demetriou, et al. [21] concluded that domains similar to the TRH1 domain existed in both the TGF-β type II receptor and the BMP type II receptor but not in any of the type I receptors. They did not explicitly delineate, however, the sequence in the BMP type II receptor that they felt was similar to the TRH1 domain. Nevertheless, the likelihood that these regions of fetuin and Spp24 participate in growth factor binding is strongly suggested by surface plasmon resonance binding studies with synthetic peptides the sequence of which were based on the TRH1 domains of fetuin and Spp24. Demetriou, et al. [21] tested the interaction of a peptide based on the TRH1 domain of fetuin (CDIHVLKQDGQFSVLFTKCD) and reported that it bound to rhBMP-2 with a modest K_D_ of 2.4 x 10^-6^ M but did not bind to TGF-β1 (“no binding” defined as K_D_ >1 x 10^-5^ M). On the other hand, a peptide based on the corresponding region within the hTGF-β receptor type II (CVAVWRKNDENITLETVCHD) bound TGF-β1 with a modest K_D_ of 1 x 10^-6^ M but did not bind rhBMP-2 (“no binding” defined as K_D_ > 1 x 10^-5^ M) [21]. Similarly, Behnam, et al. [15] tested the interaction between a peptide based on the TRH1 domain of bovine Spp24 (CRSTVRMSAEQVQNVWVRC) and rhBMP-2 and reported a modest affinity as reflected in a K_D_ of 3 x 10^-5^ M. Note that this value for the K_D_ is higher, which is to say, reflects a lower affinity, than the limit for “no binding” employed by Demetriou, et al. [21]. However, the same group subsequently reported substantially higher affinities for this peptide and a number of growth factors from the TGF-β family [18]. The differences in reported values were attributed to greater accuracy in determining the concentration of the peptides, which are very poorly soluble in aqueous solution. Precise measurements of concentration are critical for the kinetic calculations associated with SPR analyses. Specifically, the K_D_ values for the interaction between the peptide based on the TRH1 domain of Spp24 (called BMP Binding Peptide or BBP) and the growth factors were: rhBMP-2(K_D_ = 53.3 nM); rhOP-1 (also called BMP-7) (K_D_ = 11.6 nM); rhTGF-β (K_D_ = 68 nM); and rmGDF-5 (recombinant murine growth and differentiation factor-5) (K_D_ = 777 nM) [18].

The situation is made more complex by differences between the TGF-β receptors and the BMP receptors. While TGF-β binds with high affinity to the type II receptor and not at all to the isolated type I receptor, BMP binds with high affinity to the type I receptor [25] though this high affinity binding may require the simultaneous presence of the type II receptor [26]. The degrees of similarity between the TRH1 domains of fetuin or Spp24 and TGF-β or BMP-2 are relatively small. (In fact, in comparisons with the “blast” tool (www.NCBI.nlm.nih.gov), the following pair-wise sequence comparisons (Figure 5) gave results of “no significant similarity”: bovine fetuin TRH1 vs. hTGF-β R type II; bovine Spp24 TRH1 vs. hTGF-β R type II; human Spp24 TRH1 vs. hTGF-β R type II; human Spp24 TRH1 vs. human BMP R type II.) The negative conclusion of Demetriou, et al. [21] notwithstanding, it is not unreasonable, therefore, to examine the BMP receptor type I for a region similar to the TRH1 domain of Spp24. And, in fact, a domain does exit in the putative binding region of the BMP receptor type Ia and type Ib molecules [27,28] that does resemble, somewhat, the TRH1 domain of Spp24. At this time it can only be concluded that the exact structural nature of the interaction between Spp24 and members of the TGF-β family has not been precisely defined but that the TRH1 domain participates in some manner.

Our studies have shown that TGF-β binds to all four of the Spp24 forms. The pattern is, as in the case of BMP-2, that the full-length form is bound with significantly greater affinity. This suggests that the C-terminus of the molecule is required for optimal binding either through allosteric changes of the binding site or through provision of a second binding site. As discussed in detail above, it should be emphasized that the exact nature of the TGF/BMP binding site of Spp24 has not been defined by physico-chemical studies, and the binding of growth factors to aspects of the Spp24 molecule other than the TRH1 domain, most notably the C-terminus, has not been tested. The affinity of TGF-β for full-length Spp24 is less than the affinity of BMP-2 for full-length Spp24 but, the values are not greatly different. On the other hand, the affinity of TGF-β for each of the three smaller size forms of Spp24 is significantly less than that of BMP-2 (Table 1).

The inhibition of the osteogenic activity of BMP by Spp24 has been demonstrated in a number of different model systems. Parenthetically, it is interesting that Spp24 is inhibitory of BMP-2 mediated bone formation because the 18.1 kD form was isolated during efforts to characterize the active component of Urist’s “BMP/NCP” [15] and, furthermore, another early investigator published the sequence of another allegedly osteogenic protein the sequence of which was nearly identical to that of Spp24 (that had not been described at the time) [29]. In female transgenic mice in which full length Spp24 was expressed under the control of the osteocalcin (OC) promoter, femoral bone mineral density (BMD) was reduced by about 10% at three months and about 6% at 8 months [19]. It was not unexpected that the inhibition was limited to female transgenic mice since OC is expressed at higher levels in female mice. In the mouse hindquarter ectopic bone formation model, ectopic bone formation by 5 μg of rhBMP-2 was completely inhibited by co-implantation with 2.5 mg of full-length Spp24 but not affected by 0.05 mg of Spp24 [19]. On the other hand, when various doses of the different forms of Spp24 were co-implanted with 5 μg of rhBMP-2 in the assay, the different forms imparted different degrees of inhibition [16]. At the highest dose of Spp (2.5 mg), Spp24 completely inhibited BMP-2 induced bone formation, whereas Spp18.1 and Spp16.0 inhibited bone formation by about 30% and Spp14.5 inhibited bone formation by about 70% [16]. In a rat model of BMP-2 enhanced spine fusion, Spp24 reduced “bone area” by a much greater degree than did “spp18.5” (Spp18.1) [20]. In that study the “manual palpation score” (0 to 7) was 7.0 for 10 μg of BMP-2 alone, 6.0 for BMP-2 plus spp18.5, and 1.5 for BMP-2 plus full-length Spp24 [20]. Therefore, it appeared that Spp24 is a more potent inhibitor of BMP-2 activity than is Spp18.1

*In vivo*, TGF-β2 induced bone formation when it was injected into the periosteum of newborn rats as reported previously by other investigators [17]. At the concentrations that we employed, roughly a 30-fold molar excess, Spp24 inhibited the TGF-β2 induced bone formation but Spp14.5 did not. These results are similar to our previously reported findings with respect to BMP-2 in which we demonstrated that full-length Spp24 more strenuously inhibited the activity of BMP than did the smaller forms of Spp24 [16,20]. In general, we propose that Spp24 binds to TGF-β2 and inhibits its activity through a mechanism of extracellular sequestration. Specifically, we would hypothesize that the disparity between the effects of Spp24 and those of Spp14.5 relate to the greater affinity of Spp24 for TGF-β2 compared with that of Spp14.5

As outlined above, it appears that the Spp24 molecules and members of the TGF-β family of cytokines interact through binding of the growth factors to the receptor-like TRH1 domain. There is, however, a significant amount of information, as discussed above, that suggest that binding of growth factors to the TRH1 domain is a less than complete explanation for this interaction. Certainly, the data presented here and our previously reported data pertaining to the interactions of Spp24 proteins and BMP-2 strongly suggest an important role for a segment of the Spp24 molecule located near the C-terminus in the binding of growth factors to Spp24. The review of the sequence similarities presented above suggests that a β-pleated sheet/turn/β-pleated sheet motif (formally, a hairpin β motif) is involved in part of the binding domain. However, while an oxidized cyseine-cysteine bond is absolutely required for binding of short peptides with TRH1-like sequences to the pertinent growth factors, this is not the case for the full length protein. Furthermore, there appear to be very non-stringent requirements for specific amino acids in the binding domain. In summary, a thorough definition of the nature of the interaction between Spp24 and TGF-β or BMP will require comprehensive physico-chemical studies.

Of interest is the observation that Spp24 and Spp14.5 may drive osteogenic differentiation in preference to chondrogenic differentiation (Figures 3-4). The histological examination of the ectopic bone masses formed in response to TGF-β2, alone or in combination with Spp24 or Spp14.5, consisted mostly of bone and cartilage. However, there was a paucity of cartilage in the specimens from the TGF-β2 plus Spp24 or Spp14.5 treatment groups. Joyce, et al. [17] reported that injections of “low dose” (20 ng/day) induced the formation of predominantly (about 78%) intramembranous bone whereas injections with "high dose" (200 ng/day) TGF-β2 induced the formation of mostly (80%) cartilage. This is a significant finding because previous studies have also demonstrated that the kinetics of the exposure of mesenchymal progenitors to BMPs can influence whether intramembranous or endochondral bone formation predominates [30,31]. Therefore, it is possible that Spp24 and Spp14.5 affects the exposure of mesenchymal progenitors to TGF-β2 in such a way as to favor osteogenic differentiation over chrondrogenic differentiation.

In summary, we have demonstrated that Spp24 and the three natural proteolytic products of Spp24 (Spp18.1, Spp16.0, and Spp14.5) bind TGF-β. While the affinity of this interaction is about an order of magnitude less than that of the interactions between the Spp24 proteins and BMP-2, the affinity is still sufficient for the form with the greatest affinity, full-length Spp24, to inhibit the bone inducing activity of TGF-β. The most truncated of the common forms, Spp14.5, did not inhibit the bone inducing activity of TGF-β. This disparity in the difference of inhibiting potential is distinctly different than the case of the Spp24 proteins and BMP-2 though the bioassays are obviously quite different. The observed differences in the binding of different forms of Spp24 to TGF-β and to BMP and the associated differences in biological effects do have several important ramifications. First, the fact that two independent investigators decades ago reported “osteogenic proteins” that appear to be one of the forms of Spp24 remains an enigma. Secondly, the concentrations and relative proportions of the various forms of Spp24 in the active compartment of the bone environment must impose controls on the absolute and relative amounts of biologically active (not “latent”) TGF-β and BMPs. These absolute and relative amounts, in turn, govern the balance of proliferation and differentiation in bone undergoing growth, repair, or turnover. Thirdly, these differences can be exploited to engineer therapeutic molecules that affect growth factor activity on a spectrum from inhibition (sequestration) to enhancement (slow release).
